# Decoding amino acid metabolism in hepatocellular carcinoma: critical nodes and novel therapeutic strategy

**DOI:** 10.3389/fonc.2026.1806160

**Published:** 2026-06-17

**Authors:** Jiayi Zhang, Yiling Zhu, Guiyan Liu, Mengting Liao, Jianmin Wu, Chao Chen, Mengmeng Guo, Yuanye Jiang, JuanJuan Zhao, Lin Xu

**Affiliations:** 1 Key Laboratory of Cancer Prevention and Treatment of Guizhou Province, Zunyi, Guizhou, China; 2 Department of Immunology, Zunyi Medical University, Zunyi, Guizhou, China; 3 Department of Gastroenterology, Putuo Hospital, Shanghai University of Traditional Chinese Medicine, Shanghai, China; 4 Collaborative Innovation Center of Tissue Damage Repair and Regeneration Medicine of Zunyi Medical University, Zunyi, Guizhou, China

**Keywords:** amino acid metabolism, arginine, glutamine, hepatocellular carcinoma, intervention, tryptophan

## Abstract

Amino acid metabolic reprogramming is one of the crucial features of metabolic remodeling in HCC. Accumulating evidence indicates that specific amino acid metabolisms play essential roles in promoting HCC cell proliferation and the development of drug resistance. Notably, recent studies have demonstrated that regulating the intake of particular amino acids can significantly enhance the efficacy of clinical interventions for HCC, suggesting that amino acid metabolism is emerging as a promising target for therapeutic strategies. However, some key scientific questions remain unresolved, including the intrinsic relationships between the metabolism of different amino acids and their precise roles in HCC progression. In this article, we systematically review the molecular and cellular mechanisms underlying amino acid metabolic reprogramming, focusing on glutamine, arginine, and tryptophan, and explore their potential interconnections in HCC. Additionally, we update recent advances in amino acid metabolism and clinical management of HCC. Importantly, we discuss key challenges that must be addressed in future research, aiming to provide a theoretical foundation for developing novel clinical interventions targeting amino acid metabolic reprogramming in HCC.

## Highlights

- Amino acid metabolic reprogramming in HCC is closely related to its biological behaviors (e.g., growth, proliferation, and migration).
- There are complex metabolic interrelationships among different amino acids within HCC.
- Targeting amino acid metabolism is a promising strategy for the clinical treatment of HCC.

## 1 Introduction

Hepatocellular carcinoma (HCC) accounts for 75% to 85% of primary liver cancers and is the third leading cause of cancer-related deaths worldwide. The five-year relative survival rate for patients is approximately 18% (1). Notably, in recent years, both the incidence and mortality rates of HCC have increased (2). HCC is often diagnosed at an advanced stage because its early phases are frequently asymptomatic, limiting the effectiveness of therapeutic interventions (3). For certain individuals with early-stage HCC, surgical resection and liver transplantation remain the preferred treatment options. However, systemic therapies are primarily employed to prolong survival in patients with advanced or unresectable HCC (4). Among these systemic approaches, targeted therapies and immunotherapies have emerged as promising strategies, yet their efficacy remains limited by drug resistance and heterogeneous patient responses. Despite modest progress in developing treatment plans, there remains an urgent need to discover more effective therapeutic modalities and reliable predictive biomarkers that can guide personalized treatment and improve clinical outcomes. Elucidating the fundamental biological mechanisms that drive HCC initiation and progression is therefore essential for identifying novel druggable targets and developing more precise diagnostic tools.

HCC initiation and progression are now recognized to be significantly influenced by the reprogramming of amino acid metabolism. Beyond their primary role as protein building blocks, amino acids can be oxidized to replenish cellular ATP stores and supply the carbon and nitrogen necessary for *de novo* nucleotide synthesis (5). Malignant hepatocytes redirect multiple anabolic and catabolic pathways to enhance metabolic flux, as the tumor microenvironment simultaneously imposes high bioenergetic demands while restricting nutrient availability, ensuring continuous biomass and energy production. Increasing evidence indicates that HCC cells obtain most of their carbon and nitrogen precursors through reprogrammed amino acid metabolism (6, 7). To date, several therapeutic strategies targeting amino acid metabolism have been explored in preclinical and clinical settings, including: (i) dietary restriction or enzymatic depletion of specific amino acids; (ii) pharmacological inhibition of key metabolic enzymes; (iii) blockade of amino acid transporters; and (iv) modulation of downstream signaling pathways. These approaches aim to exploit the metabolic vulnerabilities of HCC cells while minimizing collateral damage to normal tissues. Therefore, we focus on exploring amino acid metabolism associated with HCC, aiming to inform the future development of amino acid metabolism-based strategies as novel targets for early clinical diagnosis and therapy of HCC.

## 2 Amino acid metabolism in HCC

**Figure 1:**
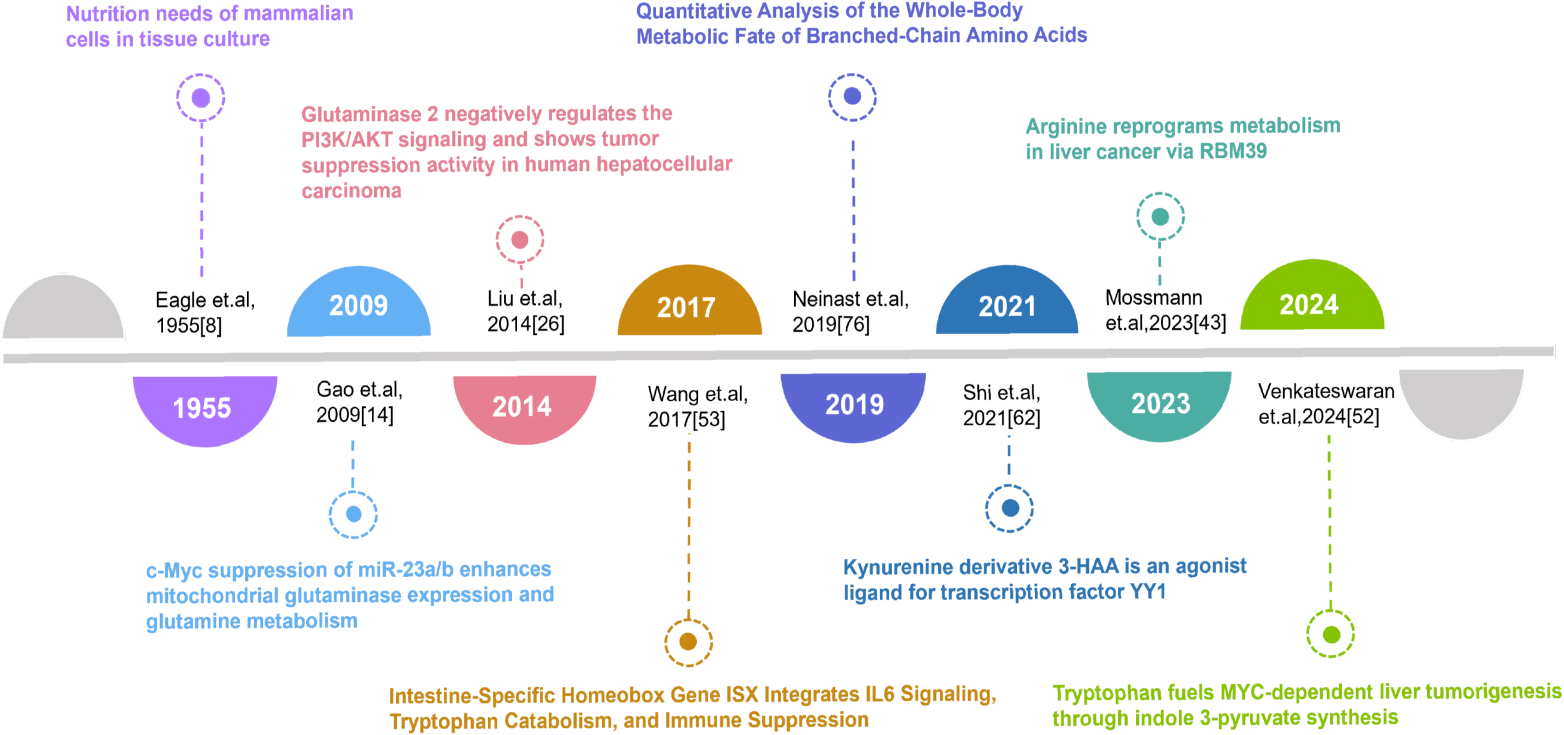
Overview of the major progression in amino acid metabolism in HCC.

The critical role of amino acid metabolism in determining the fate of cancer cells has been increasingly recognized since Eagle’s seminal 1955 discovery that tumor cell viability decreases under conditions of specific amino acid deprivation (8). More recent research has demonstrated that the reprogramming of amino acid metabolic pathways actively drives the development, proliferation, and migratory capacity of HCC cells (5–7) (**Figure 1**), facilitated by advances in metabolomics and transcriptomics. One characteristic that distinguishes HCC is its metabolic reprogramming, which reflects a profound reorganization of the cellular regulatory environment. Therefore, a mechanistic understanding of abnormal amino acid metabolism in HCC may reveal therapeutic vulnerabilities that were previously unidentified, offering new avenues for intervention within the complex phenotypic landscape of tumor heterogeneity.

### 2.1 Glutamine metabolism

**Figure 2:**
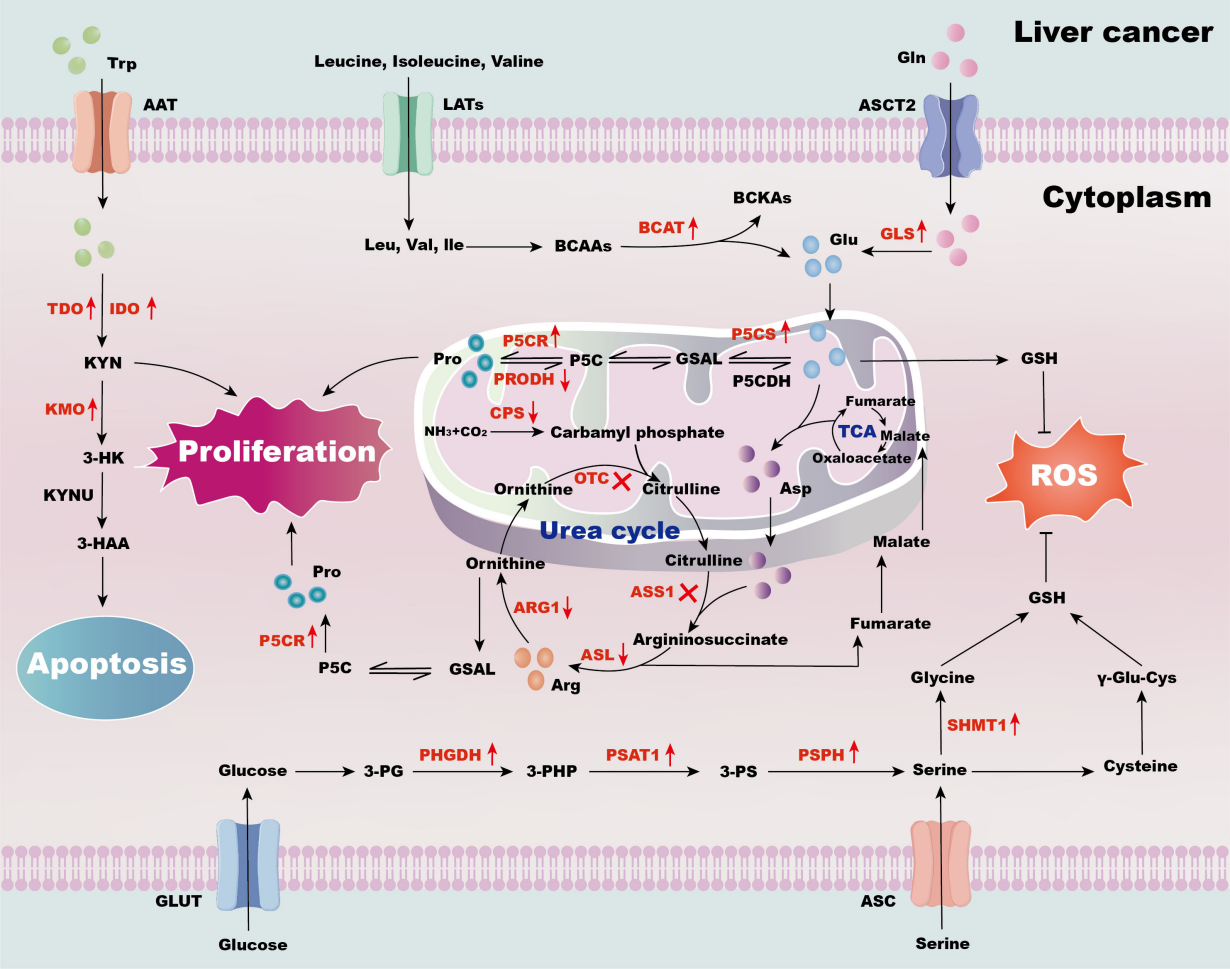
Amino acid metabolism in hepatocellular carcinoma cells. The metabolism of glutamine, arginine, tryptophan, proline, serine, and branched-chain amino acids in hepatocellular carcinoma cells. An overview of key amino acid metabolic pathways and associated critical molecules. Red arrows and crosses indicate the upregulation and blocked-expression of key enzymes in these metabolic pathways, respectively. Further details are provided in the text.

Glutamine (Gln), the most abundant free amino acid in the human body, is essential for several biological functions, including cell division, energy metabolism, antioxidant defense, and cell communication (9). Glutamine synthetase (GS) catalyzes the synthesis of Gln from glutamate and ammonia (generated *de novo*), whereas glutaminase (GLS) catalyzes the hydrolysis of glutamine back to glutamate. The tricarboxylic acid (TCA) cycle is supported by mitochondrial glutamate anaplerosis, which helps maintain the production of ATP and NADPH (10). The primary pathway for Gln absorption is the alanine-serine-cysteine transporter-2 (ASCT2/SLC1A5), which is consistently overexpressed in HCC. Gln facilitates (i) NADPH regeneration through mitochondrial metabolism, (ii) glutathione (GSH) synthesis to scavenge reactive oxygen species (ROS), (iii) *de novo* biosynthesis of nonessential amino acids (NEAAs), purines, pyrimidines, and lipids for biomass expansion, and (iv) signal transduction cascades that promote proliferation following ASCT2-mediated uptake (9). During hepatocarcinogenesis, glutamine-metabolizing enzymes exhibit isoform-specific expression patterns that are selectively reprogrammed. Mammalian cells encode two glutaminase isoforms: glutaminase 1 (GLS1) and glutaminase 2 (GLS2). GLS1 is significantly upregulated as the pre-malignant liver progresses to HCC, whereas GLS2 is often repressed or functionally replaced by GLS1 (11) (**Figure 2**). Additionally, GS ligates glutamate and ammonia to regenerate Gln, creating an endogenous nitrogen reservoir that supports HCC biomass accumulation and proliferation. More importantly, GS expression shows an increasing trend in HCC (12).

In terms of the expression mechanisms of related metabolic enzymes, recent studies have demonstrated that HIF-1α, Myc, Rb, and miRNAs can regulate the expression of GLS1, thereby influencing the biological behavior of HCC cells (13). Research has revealed that in hypoxic cells, hypoxia-inducible factor-1α (HIF-1α) directly binds to the GLS1 gene, increasing GLS1 mRNA and protein expression. This, in turn, promotes glutamine metabolism in tumor cells, thereby driving tumor cell proliferation (14, 15). Notably, downstream glutamine metabolites, such as hydroxyproline, can inhibit the hydroxylation of HIF-1α and prevent its binding to tumor suppressor proteins under hypoxic conditions. This inhibition leads to increased HIF-1α expression, which further promotes the development of HCC (16). Furthermore, the stabilization of c-Myc through methylation-mediated inhibition of ubiquitin-proteasome degradation leads to GLS1 upregulation, enhanced glutamine metabolism, and increased proliferation and chemotherapy resistance (17). Similarly, c-Myc can induce ASCT2 transcription, promote GLS1 expression and glutamine uptake, thereby enhancing cell proliferation and driving glutamine dependency (18). Retinoblastoma (Rb) enhances glutamine uptake and anaplerosis by increasing GLS1 activity (19). The liver-specific tumor suppressor miR-122 is downregulated in HCC, leading to derepression of GLS1 and consequently promoting cellular proliferation (20). Conversely, GLS2 has been identified as a target gene of the tumor suppressor protein p53 (21). In HCC, although this gene functions as a tumor suppressor gene, its expression level is significantly reduced due to the epigenetic silencing mechanism (especially promoter methylation), which inhibits the expression of GLS2 (22, 23). Zhang et al. demonstrated that GLS2 directly binds to the small GTPase Rac1, antagonizing its pro-metastatic activity and thereby inhibiting HCC progression (24). Although GLS1 and GLS2 both function as glutaminases, the controversial observation that they exhibit opposite effects in HCC is thought to be potentially related to tissue specificity, which requires further in-depth investigation in the future.

**Figure 3:**
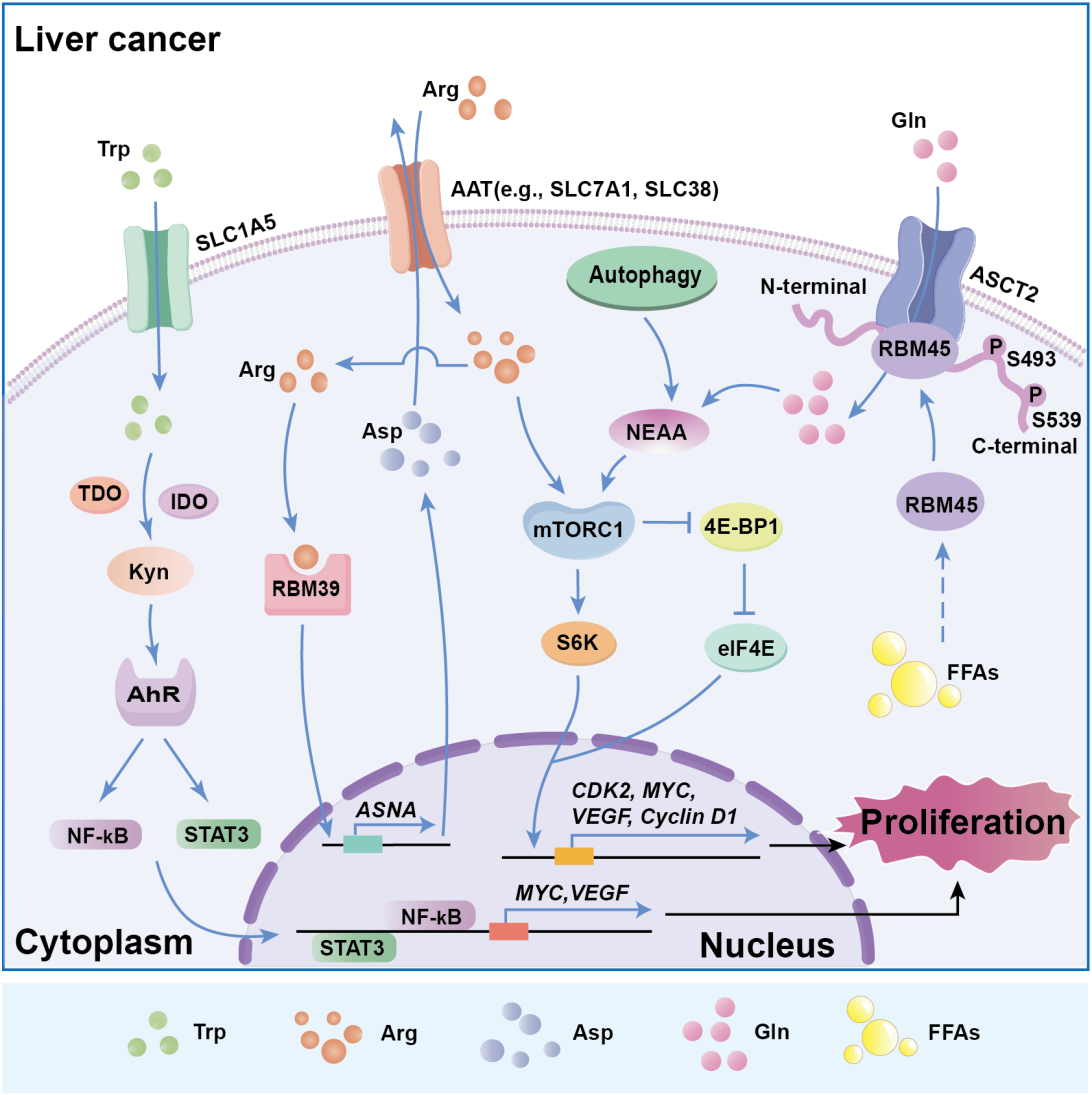
Molecular mechanisms of reprogramming of amino acid metabolism in hepatocellular carcinoma. TDO, IDO, Kyn, and AhR promote the proliferation of hepatocellular carcinoma (HCC) cells by activating the STAT3 and NF-κB pathways, thereby enhancing the expression of c-Myc and VEGF. Arginine binds to RNA-binding motif protein 39 (RBM39), which regulates the expression of metabolic genes (e.g., ASNA) to maintain high arginine levels and support oncogenic metabolism. Additionally, arginine activates mTORC1, further promoting the proliferation of HCC cells. Stimulation by free fatty acids (FFAs) increases the levels of RNA-binding motif protein 45 (RBM45), which enhances the stability of ASCT2 through phosphorylation, thereby contributing to HCC development. During amino acid starvation, glutamine cooperates with autophagy to reactivate mTORC1, which promotes translation by regulating the p70S6K/RPS6 and 4EBP1/eIF4E signaling cascades. This, in turn, facilitates the expression of genes related to cell cycle progression and proliferation (e.g., CDK2, MYC, VEGF, Cyclin D1), ultimately accelerating HCC progression.

In HCC, the differential effects of GLS1 and GLS2 on tumor cell energy metabolism are primarily attributed to their distinct roles in glutamine metabolism. GLS1 supports the continuous operation of the tricarboxylic acid (TCA) cycle and GSH synthesis by catalyzing the conversion of glutamine to glutamate, thereby providing energy and enhancing resistance to oxidative stress in HCC cells. Studies have demonstrated that GLS1 promotes HCC cell proliferation through activation of the AKT/GSK3β/Cyclin D1 signaling pathway (25). Furthermore, inhibition of GLS1 leads to the accumulation of ROS, which suppresses the nuclear translocation of β-catenin. This, in turn, reduces the expression of stemness-associated genes and diminishes the stem cell-like properties of HCC cells (11). Conversely, GLS2 suppresses HCC cell proliferation by negatively regulating the PI3K/AKT signaling pathway (26). Research indicates that GLS2 stabilizes Dicer via the ubiquitin-proteasome system, thereby promoting the maturation of miR-34a. Mature miR-34a suppresses Snail expression, reducing the invasive capacity of hepatocellular carcinoma cells and inhibiting the epithelial-mesenchymal transition process (27). Meanwhile, Suzuki et al. further discovered that GLS2 contributes to iron deprivation by regulating glutamine catabolism, thereby enhancing its tumor-suppressive effects (28). In addition, recent studies have revealed that RNA-binding motif protein 45 (RBM45) phosphorylates and stabilizes ASCT2, resulting in increased glutamine uptake and promoting the progression of HCC (29). Additionally, recent findings indicate that free fatty acid (FFA)-mediated upregulation of RBM45 activates the PI3K/AKT/mTOR signaling pathway, thereby accelerating the progression of HCC (30). It is worth noting that increasing evidence indicates that, in addition to the RTK/PI3K/PTEN/AKT cascade capable of activating mTOR, glutamine can also modulate the mTOR signaling pathway to influence hepatocellular carcinoma progression. For instance, recent studies reveal that during prolonged amino acid starvation, glutamine collaborates with autophagy to reactivate mTORC1 by being converted into glutamate and subsequently transaminated into nonessential amino acids (NEAAs), thereby promoting HCC cell proliferation (31). Furthermore, mTORC1 enhances the transcription of genes related to cell cycle progression and proliferation—such as CDK2, MYC, VEGF, and Cyclin D1—through the p70S6K/RPS6 and 4EBP1/eIF4E signaling cascades, thereby promoting tumor formation in hepatocellular carcinoma (32, 33) (**Figure 3**).

Therefore, clinically applicable agents can target key enzymes involved in glutamine metabolism, as well as glutamine transporters, to interfere with disease progression. For example, combined treatment with the GLS1 inhibitor CB-839 and the ASCT2 transporter inhibitor V-9302 has demonstrated tumor-suppressive efficacy in HCC (10). In conclusion, the reprogramming of glutamine metabolism, marked by changes in the expression of various metabolic enzymes, plays a critical role in the onset and progression of hepatocellular carcinoma. Targeting this pathway offers a promising therapeutic strategy for treating glutamine-dependent hepatocellular carcinoma.

### 2.2 Arginine metabolism

Arginine (Arg) is a semi-essential amino acid that serves as a precursor for multiple metabolites influencing cancer cell growth, proliferation, invasion, immune evasion, and metastasis (34). Arg is primarily synthesized through the urea (ornithine) cycle. In this process, the rate-limiting enzyme argininosuccinate synthase 1 (ASS1) catalyzes the condensation of citrulline and aspartate to form argininosuccinate. Subsequently, argininosuccinate lyase (ASL) cleaves argininosuccinate to release arginine and fumarate (35). Arg catabolism is mediated by arginase and nitric oxide synthase (NOS), among other enzymes. NOS converts Arg to nitric oxide (NO) and citrulline, whereas arginase produces ornithine and urea (36). Ornithine is converted back to L-citrulline by ornithine carbamoyltransferase (OTC) and subsequently recycled to Arg via the ASS1/ASL pathway. Because OTC expression is primarily restricted to the liver, Arg recycling is predominantly liver-dependent (34). Additionally, arginine decarboxylase (ADC) and arginine deiminase (ADI) catabolize Arg to agmatine and citrulline, respectively; agmatine is subsequently channeled into polyamine biosynthesis (36). Extensive analyses have revealed frequent deletions of ASS1 and/or OTC in HCC, paradoxically accompanied by elevated intracellular arginine levels. This finding indicates a significant dependency of HCC cells on exogenous Arg (37, 38) (**Figure 2**). These findings establish arginine-deprivation therapy as a promising treatment strategy for HCC.

Regarding the expression mechanisms of relevant metabolic enzymes, c-Myc and HIF-1α—both regulators of glutamine metabolic enzymes—exert opposing effects on the expression of argininosuccinate synthetase 1 (ASS1). The chemotherapy drug cisplatin upregulates HIF-1α and downregulates c-Myc; these factors act as negative and positive regulators of ASS1 expression, respectively. By binding to the ASS1 promoter, they suppress ASS1 expression, thereby inducing synthetic lethality when treated with cyclopropyl-N-deimidoyl-L-arginine (ADI-PEG20) (39, 40). ASL and arginase are coordinately downregulated in HCC; notably, intracellular Arg accumulates despite the global suppression of urea cycle enzymes, highlighting its selective advantage for HCC cells (41). This paradoxical accumulation is exacerbated by the elevated expression of the arginine-selective transporter SLC7A1, which further increases intracellular arginine levels. Knockout of the SLC7A1 gene significantly suppresses the proliferation and invasive capacity of hepatocellular carcinoma cells overexpressing the hepatitis B virus X protein (42). Critically, Arg retention is reinforced by impaired polyamine synthesis: arginase 1 (ARG1) and agmatinase (AGMAT) function in parallel to convert arginine into polyamines, yet both enzymes are downregulated in HCC, allowing tumor cells to maintain high Arg levels essential for proliferation and progression. Mossmann et al. discovered that elevated Arg levels specifically bind to RBM39, upregulating the asparagine synthetase (ASNS) gene. The resulting increase in asparagine further enhances arginine uptake, creating a positive feedback loop that sustains elevated Arg levels and promotes carcinogenic metabolism (43) (**Figure 3**). Arginine deprivation activates an integrated stress response that induces quiescence in HCC cells through a general control nonderepressible 2 (GCN2)-dependent mechanism. Pharmacological inhibition of GCN2 under arginine-starved conditions induces senescence, sensitizing HCC cells to senolytic agents. Consequently, the combined treatment of arginine deprivation and GCN2 inhibition triggers apoptosis (44). Moreover, Arg starvation impairs mitochondrial function, leading to excessive ROS accumulation, genomic instability, and the death of arginine-auxotrophic cells (45). Therefore, clinically actionable therapeutics can exploit the arginine auxotrophy of hepatocellular carcinoma cells to inhibit disease progression.

### 2.3 Tryptophan metabolism

Tryptophan (Trp) is an essential amino acid that must be obtained through daily dietary intake (46). The concentration of tryptophan in the human body is influenced by dietary intake and the activity of its metabolic pathways. Its metabolism primarily occurs through three pathways: the kynurenine (Kyn) pathway, the serotonin (5-HT) pathway, and the indole pathway (47). Among these pathways, the Kyn route is the primary hepatic pathway, accounting for over 95% of Trp catabolism and producing numerous bioactive intermediates (48). Tryptophan 2,3-dioxygenase (TDO) and indoleamine 2,3-dioxygenase (IDO) initiate the cascade by converting Trp to N-formylkynurenine. Formamidase subsequently hydrolyzes N-formylkynurenine to L-kynurenine (L-KYN), which is then hydroxylated to 3-hydroxykynurenine (3-HK) by kynurenine 3-monooxygenase (KMO). Finally, kynureninase (KYNU) cleaves 3-HK to produce 3-hydroxyanthranilic acid (3-HAA) (49). TDO and IDO are the principal rate-limiting enzymes of the Kyn pathway. In HCC, elevated expression of TDO and IDO leads to intracellular accumulation of kynurenine, which subsequently promotes tumor cell proliferation through activation of downstream oncogenic signaling pathways (50, 51) (**Figure 2**). Concomitantly, increased expression of the Trp transporters SLC1A5 and SLC7A5 enhances HCC progression (52). Moreover, the intestine-specific homeobox gene ISX has recently been shown to induce IDO and TDO expression, thereby promoting HCC cell proliferation (53).

In HCC, overexpression of TDO2 and IDO significantly increases Kyn levels. Subsequently, Kyn activates the intracellular aryl hydrocarbon receptor (AhR) and stimulates interleukin-6 (IL-6) production, which in turn promotes IDO expression through STAT3 activation. This suggests that human cancer cells can sustain their IDO expression via an autocrine IDO-AhR-IL-6-STAT3 signaling loop, thereby facilitating HCC growth and survival (54). Furthermore, the tryptophan metabolite Kyn activates AhR, which subsequently triggers the STAT3 and NF-κB/TIM4 signaling pathways. This activation upregulates the expression of MYC and VEGF, promotes the proliferation of HCC cells, and induces epithelial-mesenchymal transition (EMT). Notably, glutamine and arginine also enhance MYC and VEGF expression via the mTORC1 signaling pathway, thereby facilitating HCC progression (48, 55–57) (**Figure 3**). However, Cheng et al. found that TDO inhibits cell cycle progression by increasing the expression of the cell cycle arrest proteins p21 and p27, causing HCC cells to arrest in the G1 phase, thereby suppressing their proliferation (58). Additionally, different serotonin receptors (5-HTRs) play distinct roles in the development of HCC. For instance, 5-HT binding to 5-HTR1D activates the PI3K/AKT signaling pathway, thereby upregulating FoxO6 expression and promoting the initiation and progression of HCC (52, 59). Conversely, the binding of serotonin to 5-HT1A receptors inhibits DNA synthesis in hepatocytes, thereby exerting a negative regulatory effect on the growth of hepatocellular carcinoma cells (60). Additionally, 3-HAA negatively regulates T cell activation by inhibiting calcium signaling mediated by the T cell receptor (TCR) and promotes the formation of regulatory T cells (Tregs) by stimulating transforming growth factor-β (TGF-β) (61). In contrast, 3-HAA is significantly depleted in HCC. Its exogenous restoration binds to YY1, promotes PKCζ-mediated phosphorylation at Thr398, and enhances YY1 chromatin occupancy, ultimately leading to apoptosis (62).

The aforementioned studies highlight that tryptophan and its metabolites exert context-dependent and often opposing effects in HCC. This pleiotropic nature necessitates precise modulation of tryptophan metabolism in therapeutic strategies targeting HCC. Therefore, future research must elucidate the molecular mechanisms underlying tryptophan metabolism to establish a robust mechanistic framework for precision oncology in HCC.

### 2.4 Metabolism of other amino acids in HCC

Emerging evidence indicates that additional amino acid metabolic pathways critically mediate chemoresistance, anti-apoptotic mechanisms, and the acquisition of proliferative advantages in HCC (**Table 1**). Therefore, mechanistic dissection of these ancillary metabolic circuits will elucidate the multifaceted roles of amino acid metabolism in tumor progression and identify viable therapeutic targets.

**Table 1:** The role and mechanisms of amino acid metabolism in HCC.

Amino acid	Research subject	Clinical feature	Function	Mechanism	Ref.
Glutamine	GLS1	lymphatic metastasis, TNM stage and prognosis	cell proliferation	AKT, GSK3β, CyclinD1	(25)
	GLS1 and Cancer stem cell	prognosis	cell proliferation, and epithelial- mesenchymal transition	ROS, Wnt, β-catenin	(11)
	GLS2	**–**	suppress proliferation	PI3K, AKT	(26)
Arginine	ARG1 and AGMAT	**–**	cell proliferation	RBM39	(43)
	Arg	**–**	cell proliferation	T cell, BAZ1B, PSIP1, TSN	(111)
Tryptophan	TDO2 and Kyn	prognosis	cell proliferation	IL-6, STAT3, NF-κB, and TIM4	(55)
	5-HT	prognosis, Edmondson grade, microvascular invasion, and TNM stage	cell proliferation, epithelial- mesenchymal transition, and metastasis	PI3K, AKT, and FoxO6	(52)
	3-HAA	**–**	cell apoptosis	YY1	(62)
Proline	PYCR1 and ALDH18A1	tumor grade, and predictor	cell proliferation	**–**	(64)
	P5CS and ALDH18A1	**–**	cell proliferation	HIF1α	(16)
Cysteine	BHMT and CDO1	**–**	ferroptosis	EZH2	(75)
Serine	PHGDH	prognosis	cell proliferation	ZEB1	(71)
BCAAs	BCAAs	**–**	cell proliferation	AMPK, PROX1, mTOR	(78)
Phenylalanine	GSTZ1-1	**–**	cell proliferation	NRF2, IGF1R	(82)

#### 2.4.1 Proline

Proline (Pro) is the only proteinogenic amino acid whose α-amino group is incorporated into a pyrrolidine ring and serves as a key precursor in collagen biosynthesis (63). During proline anabolism, mitochondrial glutamate is sequentially oxidized and reduced by δ-1-pyrroline-5-carboxylate synthetase (P5CS) to form δ-1-pyrroline-5-carboxylate (P5C), while ornithine can also be converted into P5C in the cytosol. Subsequently, P5C is reduced to proline by pyrroline-5-carboxylate reductase (P5CR). In proline catabolism, proline is oxidized to P5C by proline dehydrogenase (PRODH). In HCC, the transcriptional levels of the genes encoding the two rate-limiting enzymes involved in proline biosynthesis—ALDH18A1 (P5CS) and PYCR1 (P5CR)—are increased. Conversely, the activity of proline dehydrogenase (PRODH) decreases in HCC, leading to the accumulation of proline within the cells and promoting tumor cell proliferation (64) (**Figure 2**). PYCR1 has been shown to transcriptionally upregulate insulin receptor substrate-1 (IRS1), thereby activating both the PI3K/AKT/mTOR and MAPK/ERK pathways, consequently enhancing HCC growth and metastasis (65). Moreover, proline stabilizes hypoxia-inducible factor-1α (HIF-1α), which subsequently enhances glutamine anabolism and supports tumor cell growth. In contrast, pharmacological inhibition of proline metabolism sensitizes HCC cells to sorafenib, induces apoptosis, and significantly suppresses tumor growth (16). Consequently, targeting proline metabolism represents a promising therapeutic strategy for hepatocellular carcinoma treatment.

#### 2.4.2 Serine

Serine (Ser) is a non-essential amino acid that plays a crucial role in redox homeostasis and the *de novo* synthesis of nucleotides and lipids. Its intracellular levels are primarily maintained through the *de novo* serine synthesis pathway. Initially, 3-phosphoglycerate (3-PGA) is oxidized to 3-phosphohydroxypyruvate (3-PHP) by phosphoglycerate dehydrogenase (PHGDH). Next, phosphoserine aminotransferase (PSAT1) transaminates 3-PHP to 3-phosphoserine (3-PS), which is subsequently dephosphorylated by phosphoserine phosphatase (PSPH) to produce serine. Newly synthesized serine is then converted to glycine by cytosolic serine hydroxymethyltransferase 1 (SHMT1) and mitochondrial SHMT2 (66). Serine also serves as a precursor for the biosynthesis of cysteine (Cys) (67). In HCC, the expression of PSAT1, PSPH, and SHMT1 is upregulated, and the catalytic activity of PHGDH is enhanced, collectively accelerating serine metabolism to support tumor cell proliferation (68–70). PHGDH is transcriptionally activated by zinc finger E-box binding homeobox 1 (ZEB1), thereby supplying hepatocellular carcinoma cells with sufficient precursors for protein, nucleotide, and lipid synthesis, as well as essential one-carbon units necessary for tumor growth and methylation reactions (71). Analogous to glutamine metabolism, serine biosynthesis produces GSH and NADPH to maintain redox homeostasis, thereby enhancing resistance to oxidative stress and promoting cellular proliferation (68) (**Figure 2**). Furthermore, glutamine-derived α-KG and serine one-carbon metabolism exhibit synergistic epigenetic effects. Glutaminase catalyzes the conversion of Gln to α-KG, which serves as a crucial cofactor for chromatin-modifying enzymes that regulate DNA and histone methylation (72). Concurrently, serine metabolism also generates α-KG and can be converted to glutamine, creating metabolic cross-talk within the epigenetic regulatory network (73). Consequently, targeting serine metabolism represents a promising strategy for anticancer therapy.

#### 2.4.3 Cysteine

Cysteine (Cys) metabolism plays a crucial role in sorafenib resistance in HCC. Sorafenib, an established first-line therapeutic agent for HCC, faces the significant clinical challenge of acquired resistance. Inhibition of macropinocytosis has been shown to reduce cysteine uptake in HCC cells, thereby restoring sorafenib sensitivity, enhancing ferroptosis, and ultimately suppressing tumor progression (74). Moreover, enhancer of zeste homolog 2 (EZH2) is frequently overexpressed in HCC and is associated with poor patient survival. Betaine-homocysteine thiol methyltransferase (BHMT) and cysteine dioxygenase 1 (CDO1), two key enzymes involved in cysteine metabolism, are highly expressed in normal hepatocytes but are markedly downregulated in HCC. Treatment with the EZH2 inhibitor tazemetostat restores BHMT and CDO1 expression. Consequently, tazemetostat elevates intracellular ROS by reprogramming cysteine metabolism, thereby inducing cell death and enhancing chemosensitivity (75) (**Figure 2**). Therefore, targeting cysteine metabolism represents a promising therapeutic strategy for HCC, particularly in patients who are refractory to sorafenib.

#### 2.4.4 Branched-chain amino acid

Branched-chain amino acids (BCAAs)—namely leucine, isoleucine, and valine—are essential nutrients for mammals (76). BCAAs are transaminated by branched-chain amino acid transaminase (BCAT) to produce glutamate, which is subsequently amidated by glutamine synthetase (GS) to form glutamine (**Figure 2**). In HCC, BCAT expression is significantly upregulated, while downstream catabolic enzymes are downregulated, resulting in intracellular BCAA accumulation. Reducing extracellular BCAA concentrations markedly inhibits the proliferation of HCC cells *in vitro* (77). Recent studies have demonstrated that prospero-related homeobox 1 (PROX1) is highly expressed in HCC and is associated with poor patient prognosis. Mechanistically, dysfunction of the LKB1-AMPK pathway leads to the reactivation of PROX1, which maintains intracellular BCAA levels, thereby enhancing mTOR signaling and promoting tumorigenesis. Conversely, AMPK-mediated phosphorylation induces PROX1 degradation, reducing intracellular BCAA concentrations, attenuating mTOR signaling, and consequently inhibiting HCC cell proliferation (78). Notably, BCAAs also activate mTORC1 signaling in conjunction with the metabolic pathways of glutamine, arginine, and tryptophan, forming a synergistic network that promotes HCC cell proliferation (79, 80). These findings underscore the intricate crosstalk among amino acid metabolic pathways in HCC.

#### 2.4.5 Phenylalanine

Phenylalanine (Phe) is an essential amino acid that serves as a key precursor for protein synthesis. Under physiological conditions, hepatic phenylalanine hydroxylase (PAH) converts phenylalanine into tyrosine, which subsequently participates in multiple metabolic pathways (81). However, glutathione S-transferase zeta 1-1 (GSTZ1-1), a key enzyme involved in phenylalanine and tyrosine catabolism, is absent in HCC, leading to the intracellular accumulation of the oncometabolite succinylacetone (82). Phenylalanine specifically attenuates PAH activity in HCC cells, a defect implicated in hepatocarcinogenesis (83). Succinylacetone is an oncometabolite that activates nuclear factor erythroid 2-related factor 2 (NRF2) signaling through covalent modification of Cys-406 in kelch-like ECH-associated protein 1 (KEAP1). Activation of NRF2 transcriptionally upregulates insulin-like growth factor receptor 1 (IGF1R), thereby promoting anti-apoptotic signaling and accelerating HCC progression. Pharmacological inhibition of either NRF2 or IGF1R disrupts this pathway, suppressing HCC cell proliferation and tumor nodule formation (82). Consequently, targeting phenylalanine metabolism represents a promising therapeutic strategy for HCC.

## 3 Clinical application of amino acid metabolism in HCC

A comprehensive analysis of the critical roles of amino acid metabolism in HCC has elucidated how these metabolites promote tumor progression by modulating signal transduction and redox homeostasis. The identification of these metabolic nodes not only underscores the complexity of metabolic reprogramming in HCC but also highlights potential targets for therapeutic intervention.

### 3.1 Early diagnosis

Early diagnosis is hindered by the lack of overt clinical manifestations during the initial stages of HCC. Consequently, reliable biomarkers for early detection are urgently needed. An exploratory study identified elevated plasma phenylalanine as a predictor of future HCC in patients with cirrhosis (84). Quantitative metabolomics revealed that, compared to chronic hepatitis B controls, HCC patients exhibited significant alterations in serum amino acid profiles. Specifically, there were decreases in leucine, lysine, threonine, tryptophan, valine, hydroxytryptophan, and taurine, alongside an increase in phenylalanine. Furthermore, HCC was characterized by marked reductions in the tryptophan ratio, BCAA/aromatic amino acid ratio, BCAA/tyrosine ratio, Fischer ratio, and hydroxytryptophan/tryptophan ratio, accompanied by elevated tyrosine levels and increased kynurenine/tryptophan ratios (85). A subsequent study demonstrated that the combined serum metabolites phenylalanine-tryptophan (Phe-Trp) and glycocholate (GCA) outperform α-fetoprotein (AFP) in detecting AFP-negative HCC and in distinguishing small HCC (S-HCC) from high-risk cirrhotic nodules. Moreover, the Phe-Trp/GCA panel exhibits high sensitivity for preclinical HCC and, when combined with AFP, enhances the prediction of HCC risk prior to clinical diagnosis (86). Similarly, an immunohistochemical panel comprising glutamine synthetase (GS), glypican-3 (GPC3), and heat shock protein 70 (HSP70) facilitates the differentiation of well-differentiated HCC from focal nodular hyperplasia and cirrhosis (87). Collectively, these findings suggest that the aforementioned biomarkers hold significant promise for the early detection of HCC in clinical practice.

### 3.2 Treatment

A series of recent studies have demonstrated that amino acid deprivation holds significant potential for the treatment of HCC, particularly through arginine deprivation. Currently, researchers have developed and isolated four distinct arginine-catabolizing enzymes: microbial-derived arginine deiminase (ADI), recombinant human arginine decarboxylase (rhADC), recombinant human arginase 1 (rhArg1), and Bacillus caldovelox arginase (BCA) (88). A clinical trial involving a patient with unresectable HCC treated with ADI-PEG20 demonstrated a reduction in plasma arginine levels, a decrease in tumor size and serum alpha-fetoprotein levels, and no treatment-related toxicity or side effects (89). Another phase I/II dose-escalation trial found that ADI-PEG20 treatment was effective and well tolerated in a subset of HCC patients, resulting in two complete remissions, seven partial remissions, and seven cases of stable disease among 19 patients (90). Meanwhile, ADI-PEG20 selectively inhibited HCV replication in HCV-infected HCC patients and reduced HCV titers by up to 99% in 50% of HCV-1b patients, resulting in a significant improvement in liver function (91). However, a phase III study did not show a significant difference in overall survival or progression-free survival between ADI-PEG20 and placebo (92). Specifically, because ADI is of microbial origin, its immunogenicity may provoke severe neutralizing antibody responses in patients, potentially causing anaphylaxis and significantly limiting both the duration and efficacy of treatment (92). In addition, studies have shown that in certain clinical settings, the efficacy of ADI-PEG20 monotherapy is transient, primarily due to host neutralizing antibodies against the drug and the adaptive re-expression of ASS1 in tumor cells, which restores endogenous arginine synthesis and bypasses the metabolic blockade (93). Therefore, given the immunogenicity limitations of ADI-PEG20, rhARG1 has emerged as a more promising therapeutic option for hepatocellular carcinoma. Furthermore, in a phase I study, rhArg1-PEG effectively reduced arginine levels in a dose-dependent manner. The study identified 1600 U/kg as the optimal biological dose, at which 26.7% of patients achieved disease stability (94). In subsequent phase II studies, patients who experienced arginine depletion for two months or longer demonstrated significantly longer progression-free survival, and the drug was well tolerated (95). Similarly, rhArg1-PEG was used in combination with chemotherapy in previously treated HCC patients and demonstrated significant anticancer activity (96). Although clinical trials of arginine deprivation for the treatment of HCC have shown promising results, further studies are necessary to determine the optimal therapeutic regimen to enhance treatment efficacy and improve patient survival.

Analogous to arginine deprivation, blockade of glutamine uptake has shown promising therapeutic outcomes in HCC. V-9302, a competitive small-molecule antagonist of transmembrane glutamine transport, selectively and concentration-dependently inhibits SLC1A5 (ASCT2)-mediated glutamine uptake. It exhibits 100-fold greater potency than γ-L-glutamyl-p-nitroanilide and reduces cancer cell growth and proliferation while promoting cell death and oxidative stress. In xenograft mouse models, V-9302 suppresses tumor growth and sensitizes glutamine-dependent HCC cells to the glutaminase inhibitor CB-839 by inducing ROS and apoptosis (97). Recent data have revealed a novel antineoplastic mechanism of the topoisomerase I inhibitor topotecan: the downregulation of SLC1A5, leading to the inhibition of glutamine uptake in gastric cancer cells (98). Several natural products also exhibit antitumor efficacy by modulating genes or proteins involved in amino acid and glutamine metabolism. For example, berberine, a natural compound derived from herbs, can inhibit glutamine absorption, suppress the growth of liver cancer xenograft tumors, and inhibit the proliferation of liver cancer cells *in vitro* (99). JPH203, a selective SLC7A5 inhibitor, was recently evaluated in a phase I clinical trial. Among 17 patients with advanced solid tumors, six achieved either a partial response or stable disease; notably, four of these six responders had biliary tract cancer. Additionally, plasma levels of SLC7A5 substrates remained elevated (100). Encouraging phase I results have prompted an ongoing phase II trial of JPH203 in patients with advanced biliary tract cancer (101). In addition, the novel SLC7A5-directed small-molecule inhibitor QBS10072S is currently being investigated in a dose-escalation phase I study to evaluate its safety, tolerability, pharmacokinetics, and pharmacodynamics in patients with advanced malignancies, including HCC. Efficacy data are pending (101).

Significant progress has been made in treating HCC using inhibitors that target key rate-limiting enzymes involved in amino acid metabolism. For example, GLS inhibitors such as BPTES, compound 968, and CB-839 have demonstrated potential antitumor effects in HCC. Studies have shown that treatment with the GLS-specific inhibitor BPTES reduces tumor progression and prolongs survival in a Myc-driven hepatocellular carcinoma mouse model (102). However, the poor solubility of BPTES limits its clinical application. Therefore, researchers have developed several BPTES derivatives, including CB-839, to improve its solubility. In Phase I and II clinical trials of CB-839, although it demonstrated promising efficacy against triple-negative breast cancer (TNBC) *in vitro*, its therapeutic effect as a monotherapy was limited (103). Similarly, in the Phase I and Phase II clinical trials of CB-839, the combination of CB-839 with anti-PD-1 antibodies demonstrated acceptable safety in melanoma and other malignancies, such as renal cell carcinoma. However, it has not been proven that this combination produces additional anti-tumor effects beyond those achieved by immune checkpoint blockade therapy alone (104). Notably, these trials did not include patients with hepatocellular carcinoma. Therefore, research on CB-839 in combination therapies is becoming increasingly widespread in HCC. For example, the combination of the GLS inhibitor 968 with dihydroartemisinin (DHA) significantly elevates intracellular reactive oxygen species levels while reducing GSH content, thereby demonstrating synergistic antitumor effects in the treatment of HCC (105). Consistently, the combination of the GLS1 inhibitor CB-839 with V-9302, a novel inhibitor of the glutamine transporter ASCT2, demonstrated tumor-suppressive effects in HCC (10). These findings suggest that targeting glutamine metabolism through inhibitors of glutamine transporters, such as GLS and SLC1A5, may offer an effective therapeutic strategy for HCC, particularly when combined with other treatments.

### 3.3 Prognosis

Currently, research on amino acid metabolism and HCC prognosis is making significant progress. For example, GLS1, a key enzyme in glutamine catabolism, is highly expressed in HCC and is significantly associated with shorter overall survival (OS), suggesting its potential as a marker of poor prognosis (106). In addition, low baseline arginine (ARG) levels independently correlate with reduced clinical benefit rates, shorter progression-free survival (PFS), and decreased OS (107). Metabolism-related gene-based prognostic models can stratify HCC patients and guide personalized treatment strategies. For example, glutamine metabolism-associated gene (GMAG) signatures demonstrate strong predictive accuracy for HCC patient outcomes (108).

## 4 Summary and pending issues

**Figure 4:**
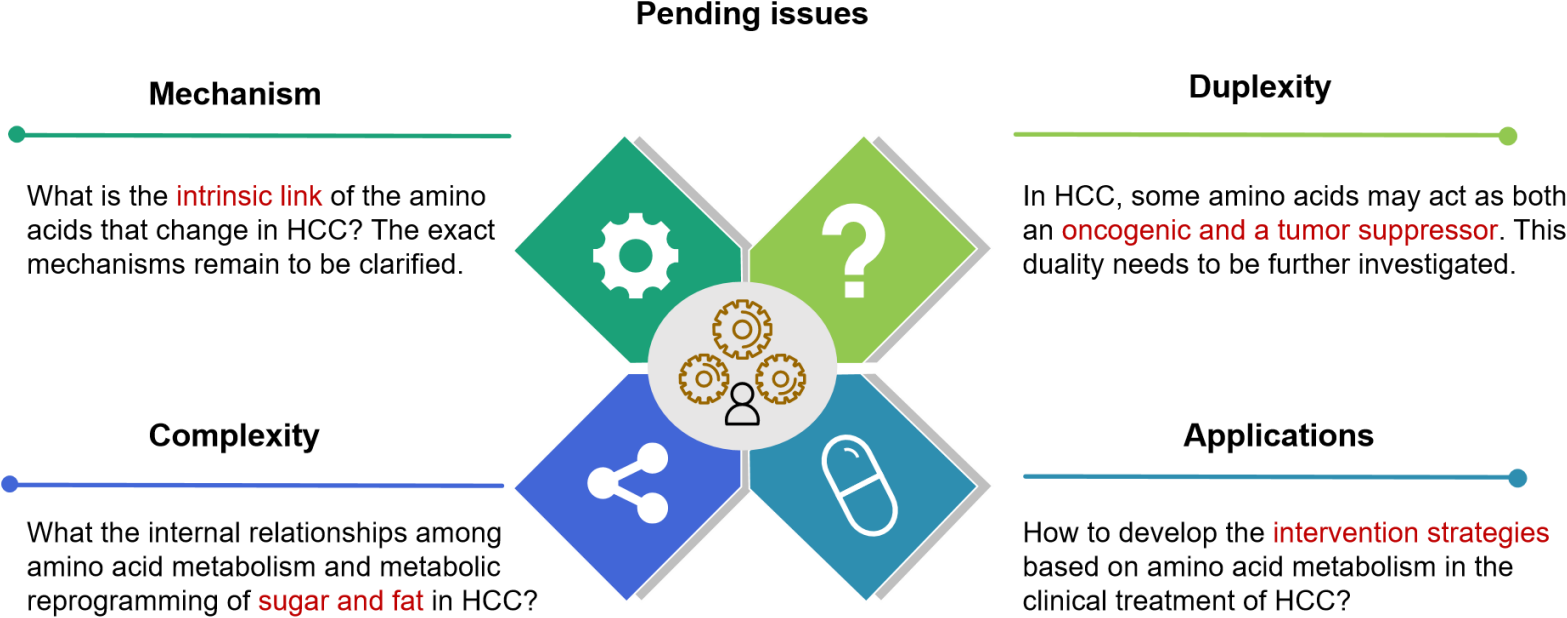
A diagram about the pending issues in amino acid metabolism in HCC.

Reprogramming amino acid metabolism has emerged as a central theme in the pathogenesis and therapeutic intervention of HCC. Recent studies demonstrate that alterations in amino acid metabolism are closely linked to HCC progression and therapeutic response, indicating that metabolism-based interventions hold significant promise. However, the diversity and complexity of amino acid metabolism in HCC—particularly the intrinsic interdependent relationships among various amino acids—pose several key unresolved challenges. Addressing these issues will provide important theoretical and technical insights for clinical treatment (**Figure 4**).

Firstly, certain amino acids in HCC exhibit complex dual effects. Specifically, these amino acids can promote tumor growth in some cases while inhibiting tumor progression in others. For example, the glutaminase isoforms GLS1 and GLS2 have opposing functions: GLS1 promotes tumor growth, whereas GLS2 acts as a tumor suppressor. Mechanistically, hypoxia-induced activation of HIF-1α upregulates GLS1, thereby enhancing HCC cell migration, invasion, and metastasis (15). Conversely, GLS2 suppresses HCC progression by negatively regulating the PI3K/AKT signaling pathway (26). The molecular determinants underlying the functional divergence between GLS1 and GLS2 remain to be elucidated. Glutamine deprivation also induces tumor cell PD-L1 expression, resulting in impaired T-cell activation. Under glutamine-limited conditions, decreased intracellular GSH attenuates Sarco/ER Ca²^+^ATPase (SERCA) activity, thereby activating the Ca²^+^/NF-κB axis and upregulating PD-L1, ultimately suppressing anti-tumor immunity (109). Conversely, glutamine metabolism inhibitors, such as JHU-083, restore anti-tumor immunity by reducing tumor-derived colony-stimulating factor 3 (CSF3). This reduction curtails the recruitment of myeloid-derived suppressor cells (MDSCs) and promotes the expansion of pro-inflammatory tumor-associated macrophages (TAMs), collectively enhancing T-cell-mediated cytotoxicity (110). Similarly, arginine deprivation suppresses both HCC cell proliferation and CD8^+^ T-cell function, thereby attenuating anti-tumor immunity and facilitating tumor progression (111). Specifically, arginine starvation selectively kills arginine-deficient tumor cells by impairing mitochondrial function, inducing excessive accumulation of ROS and causing genomic instability (45). Conversely, the bis-arginine enzyme inhibitor OATD-02 significantly enhances the anti-tumor immune response by blocking arginine degradation and increasing arginine levels in the microenvironment. This effect is evidenced by increased T cell infiltration within the tumor, expansion of CD8^+^ T cells in the draining lymph nodes, and upregulation of systemic T cell activation markers (112). Moreover, the tryptophan metabolites I3P and 3-HAA exert diametrically opposed effects in HCC (52, 62) (**Figure 2**). Therefore, this complexity suggests that the multiple effects of amino acid metabolism on both tumor cells and immune cells must be considered in an integrated manner when treating HCC.

Secondly, extensive alterations in amino acid metabolism occur in HCC, reflecting both oncogenic reprogramming and drivers of tumor growth, invasion, and immune evasion. These observations raise several fundamental questions. For example, hypoxia-inducible factor 1 (HIF-1α) and c-Myc coordinate glutamine and arginine metabolism, while proline stabilizes HIF-1α (14–16). Serine, cysteine, arginine, and glutamine metabolism all modulate ROS production (45, 68, 75, 113) (**Figure 2**). This evidence compellingly illustrates that amino acid metabolism must be understood as a dynamic, interconnected network rather than as isolated, independent changes. This raises critical questions: How are these individual metabolic pathways interconnected, and what fine-tuned regulatory mechanisms govern their crosstalk? Proline metabolism is intimately coupled to collagen synthesis; tumor cells reprogram this pathway to remodel the extracellular matrix (ECM), thereby facilitating invasion and metastasis (114, 115). By what mechanisms does amino acid metabolism shape the tumor microenvironment (TME) and consequently influence HCC behavior? Glutamine-derived α-ketoglutarate (α-KG) anaplerotically fuels the tricarboxylic acid (TCA) cycle, intersects with glycolytic intermediates, and provides carbon for energy production, biosynthesis, and lipogenesis (116, 117). How, then, does amino acid metabolism perturb lipid and glucose metabolism in HCC? What are the differences in amino acid metabolic dependencies across various HCC etiologies, such as hepatitis B virus (HBV), hepatitis C virus (HCV), alcohol-related liver disease, or non-alcoholic fatty liver disease (NAFLD)? Addressing these questions is essential for elucidating the metabolic mechanisms underlying HCC and for developing targeted therapeutic strategies. Integrating genome-wide association studies (GWAS), epigenetic profiling, transcriptomics, and public repositories such as The Cancer Genome Atlas (TCGA) will enable a system-level analysis of amino acid metabolism in HCC. Moreover, amino acid metabolomics can detect HCC earlier than conventional diagnostic methods by quantifying circulating metabolic alterations (85). However, its high cost currently prevents widespread clinical implementation, representing a significant challenge that must be addressed.

Finally, specific intervention strategies targeting amino acid metabolism in HCC, including combinations with other therapeutic approaches, remain to be thoroughly explored. Given the complex metabolic roles of amino acids in tumors and the immune system, as well as their diverse functions, simply reducing a single amino acid (such as arginine) is insufficient to control tumor growth and typically fails to achieve clinically significant therapeutic outcomes. Therefore, future efforts should focus on seamlessly integrating amino acid–targeted strategies with existing treatment modalities. Accumulating evidence indicates that the metabolism of cysteine, serine, and proline plays a critical role in drug resistance in HCC (16, 75, 118). Recent studies demonstrate that blocking cysteine metabolism induces ferroptosis in HCC cells. Mechanistically, EZH2 inhibitors reprogram cysteine-metabolic genes—including BHMT and CDO1—thereby promoting lipid ROS accumulation and ferroptotic cell death. This process sensitizes HCC cells to sorafenib, resulting in synergistic tumor suppression. Furthermore, combined dietary arginine restriction, inhibition of general control nonderepressible 2 (GCN2), and blockade of B-cell lymphoma 2 (BCL2) significantly enhance apoptosis in HCC cells (44). It is worth noting that by targeting factors such as RBM39, which bind to arginine (rather than directly reducing arginine levels), it is possible to inhibit tumor growth while avoiding T cell dysfunction (43). Furthermore, recent studies have shown that the combination of the IDO inhibitor BMS-986205 and nivolumab demonstrates manageable safety profiles in a small cohort of patients (119). Collectively, these findings underscore the therapeutic potential of rational combination therapies targeting amino acid metabolism in HCC.

In conclusion, advances in genomics, metabolomics, spatial transcriptomics, and CRISPR/Cas technologies are poised to elucidate the molecular mechanisms underlying amino acid metabolic reprogramming in HCC. These developments will deepen our understanding of HCC pathogenesis and guide the design of innovative clinical interventions.
